# Association Between ABCG1/TCF7L2 and Type 2 Diabetes Mellitus: An Intervention Trial Based on a Case–Control Study

**DOI:** 10.1155/jdr/9356676

**Published:** 2025-02-26

**Authors:** Yinxia Su, Bo Shang, Xiaoyuan Hu, Zhihao Zhang, Li Wang, Kun Luo, Hua Yao, Xiangtao Liu, Yaoqin Lu, Sheng Jiang

**Affiliations:** ^1^State Key Laboratory of Pathogenesis, Prevention and Treatment of High Incidence Diseases in Central Asia, Urumqi, Xinjiang, China; ^2^School of Medical Engineering and Technology, Xinjiang Medical University, Urumqi, Xinjiang, China; ^3^Department of Hospital Administration, The First Affiliated Hospital of Xinjiang Medical University, Urumqi, Xinjiang, China; ^4^The Clinical Medicine Research Institute, First Affiliated Hospital of Xinjiang Medical University, Urumqi, Xinjiang, China; ^5^Department of Hospital Administration, The Second Affiliated Hospital of Xinjiang Medical University, Urumqi, Xinjiang, China; ^6^School of Health Management, Xinjiang Medical University, Urumqi, Xinjiang, China; ^7^Department of Hospital Administration, Urumqi Center for Disease Control and Prevention, Urumqi, Xinjiang, China

**Keywords:** ABCG1, methylation, SNP, TCF7L2, Type 2 diabetes mellitus

## Abstract

**Background and Objective:** Type 2 diabetes mellitus (T2DM) is the result of both genetic and environmental factors. Environmental factors may contribute to the occurrence and development of T2DM by influencing epigenetic modification. The objective of this study was to explore the potential functions of two SNP-CG sites (rs7901695 of TCF7L2 and cg06500161 of ABCG1) that are most strongly associated with T2DM. Given that Uyghur population has been less studied, we conducted an intervention trial in Uyghur people to provide evidence for personalized health management of T2DM in them.

**Methods:** From May to July 2022, 320 patients with T2DM and 332 patients without T2DM were treated with dietary pagoda-based health education intervention. The demographic data were collected before intervention and basic physical biochemical indexes before and after intervention by questionnaire and physical biochemical examination. SNP typing was performed by the TaqMan-MGB probe method, and gene methylation was detected by the pyrosequencing method.

**Results:** The rs7901695 genotype difference of TCF7L2 was statistically significant between the case group and the control group (*p* < 0.05). After adjusting for covariates (smoking, alcohol consumption, exercise, fasting blood glucose (FPG), obesity, and hypertension), the genotype of rs7901695 in the TCF7L2 gene was associated with genetic susceptibility to T2DM in additive (TC vs. TT,*p* = 0.047; CC vs. TT,*p* = 0.010), dominant (*p* = 0.015), and recessive (*p* = 0.039) models. Before intervention, there were significant differences in the intake of water between the case group and the control group (*p* < 0.05). After intervention, there was statistical significance in the intake of coarse grains, fruits, aquatic products, eggs, dairy products, soy products, nuts, edible oils, and water between the case group and the control group (*p*s < 0.05). Logistic regression analysis showed that methylation of the ABCG1 gene was correlated with T2DM susceptibility after adjustment of covariable before intervention (*p* = 0.015, odds ratio (OR): 1.023; 95% confidence interval (CI): 1.004~1.041) but not after intervention. Generalized multifactor dimensionality reduction (GMDR) showed that the rs7901695 locus of the TCF7L2 gene and the cg06500161 locus of the ABCG1 gene had interaction with hypertension, dyslipidemia, abdominal obesity, and obesity and also had interaction with drinking, smoking, and exercise.

**Conclusions:** The interaction of the rs7901695 site of the TCF7L2 gene and the cg06500161 site of the ABCG1 gene with environmental factors may increase the risk of T2DM in Uyghurs. The interaction between the cg06500161 site of the ABCG1 gene and environmental factors on T2DM varied with the intervention. The cg06500161 site of ABCG1 may serve as a biomarker to evaluate the effect of T2DM interventions.

## 1. Introduction

Type 2 diabetes mellitus (T2DM) is one of the most important chronic noninfectious diseases in the world. It is a chronic hyperglycemic disease caused by insulin resistance or insufficient insulin secretion [1]. Today, T2DM has become a public health problem around the world. According to the International Diabetes Federation (IDF), the global prevalence of diabetes is estimated at 9.3% in 2019, rising to 10.2% by 2030 and 10.9% by 2045. Compared to 2017, the number of cases of T2DM increased by 1.03 times globally and by about 1.02 times in China [2]. From the 1980s, the prevalence of T2DM has been increasing year by year. The prevalence of T2DM was less than 1% in 1980 [3] but increased to 5.5% in 2001 [4] and 11.2% during 2015–2017 among adults aged 18 and above [5]. From the perspective of population distribution, the prevalence of diabetes in middle-aged and elderly people is much higher than that in other age groups [6]. T2DM is a complex chronic metabolic disease mainly caused by the combined effects of genetic and environmental factors [7]. Current studies have shown that many environmental factors such as irregular diet, high intake of sugar and salt, lack of exercise, bad living habits, and air pollution are risk factors for T2DM. Studies also have shown that T2DM is a family aggregation disease, and about 30%–70% of patients with a family history of diabetes are related to genetic factors. Since the first genome-wide association study (GWAS) on T2DM was conducted in 2007, more than 120 genes related to T2DM have been found so far [8]. In addition, environmental factors such as drinking, smoking, and diet may affect epigenetic changes and promote the occurrence and development of T2DM. Recent studies have found that epigenetics is a bridge between environmental factors and genes [9], in which DNA methylation is an important part of epigenetics, which refers to the modification of cytosine into methyl cytosine under the action of DNA methyltransferases (DNMTs). DNA methylation is a normal physiological process controlled by gene expression, but high or low DNA methylation expression will lead to the occurrence of diseases [10]. It has been reported that, in contrast to DNA sequences, DNA methylation changes dynamically over time [11]. Current research on DNA methylation and diabetes is mainly carried out from two aspects: One is to conduct genome-wide scanning for diabetes-related differential DNA methylation; the other is to use specific primers to detect diabetes-related genes with known functions in previous studies by differential DNA methylation. Current studies on gene methylation and T2DM include PPARGC1, SLC30A8, FOSL2 [12], and adenosine triphosphate–blinding cassette transporter G1 (ABCG1) [13]. Among them, ABCG1 gene methylation is considered strongly associated with T2DM. ABCG1 gene is located in chromosome 21q22.3 and is involved in lipid metabolism in the body, affecting lipid levels and leading to cardiovascular diseases and metabolic diseases as one of the members of the ABC transporter family [14]. Current studies have shown that the ABCG1 gene can be highly expressed in many tissues, such as the heart, liver, spleen, and brain. Normal expression of the ABCG1 gene can protect blood vessels, but low expression of the ABCG1 gene can lead to a disorder of cholesterol metabolism and impaired insulin secretion [15]. In addition, the methylation of the ABCG1 gene promoter region will cause lipid metabolism disorder and inhibit the protective effect of blood vessels [16]. Studies have also shown that hypermethylation of the ABCG1 gene is closely associated with the risk of coronary heart disease [17] and cerebral infarction [16]. The methylation level of the ABCG1 gene was found to be positively correlated with the risk of T2DM in the United Kingdom, the Netherlands, and Finland.

In the studies on T2DM and gene polymorphism, the polymorphism of transcription factor 7 analogue 2 (TCF7L2) was the strongest associated with T2DM. TCF7L2 gene consists of 14 exons and 13 exons, located in chromosome 10q25.3 [18], and is mainly expressed in human islet beta cells and adipose tissue [19] TCF7L2 has been reported to be an important risk gene for T2DM, with several SNPs associated with 43%~50% increases of diabetes risk, but its pathogenesis in T2DM has not been fully elucidated [18]. It has been reported that the decreased insulin secretion function in diabetes is also associated with the variation of the TCF7L2 gene, and the TCF7L2 gene is associated with the pathogenesis of T2DM, possibly due to the decreased function of pancreatic *β* cells. A study by Ding et al. showed that the rs7903146 locus of the TCF7L2 gene was significantly associated with T2DM in Caucasian, South Asian, East Asian, and other ethnic groups [20]. A meta-study showed that four variants of the TCF7L2 gene (rs7903146, rs7901695, rs12255372, and rs11196205) were associated with T2DM [18]. The rs7903146 site of the TCF7L2 gene is the most studied site and is strongly correlated with T2DM. However, this site is located in the intron rather than the CpG-SNP site, and rs7901695 is both the risk SNP and CpG site of T2DM [21]. Research indicates that there is a strong linkage disequilibrium (LD) (*R*^2^ = 1) between rs7903146 and rs7901695 [22]. Therefore, this study will include rs7901695 located in the exon as the research subject.

In addition, current studies have shown that the implementation of intervention has significant changes in various indicators of T2DM. Nowadays, community T2DM health management level is improving with the development of community health service centers. Dietary health education interventions for T2DM are also gradually mature. Studies have shown that the fasting blood glucose (FPG) and 2 h postprandial blood glucose of T2DM patients are significantly lower than before treatment after 1 year's comprehensive intervention of diet, psychology, exercise, and drugs, and the patients' self-management level and understanding of the disease are significantly improved compared with before intervention. A study on comprehensive health education intervention, such as diet, exercise, and medication, showed that T2DM patients' knowledge of diabetes was significantly improved after health education, as well as their blood sugar level [17]. Rönn et al. used epigenome-wide association studies (EWASs) to study the influence of exercise intervention on gene methylation level after 6 months and found that methylation levels of five sites in the intron region of TCF7L2 were statistically different before and after the intervention (*p* < 0.001) [23]. In a 2-year study of lifestyle interventions (exercise and diet), diabetes-related TCF7L2 alleles were associated with weight loss caused by lifestyle interventions, and the diabetes-inducing effects of rs7903146 and rs12255372 on TCF7L2 were mitigated by lifestyle interventions [24]. The above studies indicate that diet, exercise, and psychological and other interventions for diabetic patients can alleviate the course of the disease and reduce other risk factors.

The prevalence of T2DM is significantly different among different ethnic groups due to the differences in geographical location, lifestyle, and eating habits. Located in the western part of China, Xinjiang is an important geographical location along the Silk Road. Moyu County in Hotan Prefecture of Xinjiang is a gathering place of Uyghur people, with its unique living and eating habits. The dietary pattern of “refined grains and meat, like beef and mutton,” and the dietary pattern of “oil and salt” were formed by the Uygur people under the long river of history and culture [25, 26]. And the traditional lifestyle of Uygur people includes farming, animal husbandry, and handicraft activities, but with the development of urbanization and modernization, which is associated with T2DM, their lifestyle has gradually changed into sedentary and lack of physical activity [27–29].

Given that the SNP and DNA methylation of TCF7L2 and ABCG1 have been seldom studied in Uyghur and the Uyghur in Moyu County, Hotan, who are the permanent Uyghur residents and have lived there for generations and have not intermarried with other ethnic groups, are an ideal population for studying the genetic factors of T2DM, we conducted an intervention study based on a case–control study among the Uyghur population in there to explore the changes of two genes' methylation before and after the health education intervention and further corroborate the relationship between gene methylation and T2DM, which also laid the foundation for future research.

## 2. Methods

### 2.1. Subjects

A total of 320 Uygur T2DM patients (male: 158; female: 162) aged 55.50 ± 12.00 years old were selected from Moyu County People's Hospital and Rockman Hospital as the case group.

Inclusion criteria include, according to the Diagnostic Guidelines for Type 2 Diabetes in China, diagnostic criteria: fasting plasma glucose ≥ 7.0 mmol/L or 2 h blood glucose ≥ 11.1 mmol/L after oral glucose tolerance test (OGTT) or taking hypoglycemic drugs or having a history of diagnosed diabetes. Exclusion criteria include Type 1 diabetes mellitus, gestational diabetes mellitus and other types, malignant tumors, chronic infectious diseases, autoimmune diseases, psycho-neurosystemic diseases, and serious heart and kidney diseases.

The control group included 332 subjects (male: 165; female: 167) free of diabetes, aged 55.00 ± 13.00 years, who took physical examinations in the hospitals during the same period. Inclusion criteria include fasting plasma glucose < 7 mmol/L and 2 h glucose < 7.8 mmol/L after OGTT. Exclusion criteria include previous diagnosis of T2DM or physical examination diagnosis of T2DM, malignant tumors, chronic infectious diseases, psycho-neurosystemic diseases, autoimmune diseases, and serious heart and kidney diseases. The study protocol was approved by the Medical Ethics Committee of Xinjiang Medical University (Approval No. 20190225-86). Written informed consent was obtained from all participants prior to their inclusion in the study.

### 2.2. Definition and Measures

#### 2.2.1. Disease Diagnostic Criteria

(1) The diagnostic criteria of T2DM refer to Chinese Guidelines for Prevention and Treatment of Type 2 Diabetes (2020 Edition): FPG ≥ 7 mmol/L, OGTT 2 h blood glucose ≥ 11.1 mmol/L, and HbA1c ≥ 6.5% have been diagnosed with T2DM or are taking hypoglycemic medications. (2) The diagnostic criteria of obesity refer to Chinese Adult Guidelines for Prevention and Control of Overweight and Obesity (Excerpt) [30]: Body mass index (BMI) ≥ 28 kg/m^2^ is considered as obesity; female waist circumference (WC) ≥ 85 cm and male WC ≥ 90 cm were considered abdominal obesity. (3) The diagnostic criteria for dyslipidemia refer to Chinese Adult Guidelines for the Prevention and Treatment of Dyslipidemia (2016 Revision) [31]: Hypertriglyceridemia was defined as triglyceride (TG) ≥ 1.7 mmol/L; hypercholesterolemia: total cholesterol (TC) ≥ 5.2 mmol/L; high-density lipoprotein (HDL) cholesterol (HDL‐C) < 1.0 mmol/L; and low-density lipoprotein (LDL) cholesterol (LDL‐C) ≥ 3.4 mmol/L. In the above cases, one is judged as dyslipidemia. (4) The diagnostic criteria for hypertension refer to Chinese Guidelines on Hypertension Prevention and Treatment (2018 Revision) [32]: systolic blood pressure (SBP) ≥ 140 mmHg and/or diastolic blood pressure (DBP) ≥ 90 mmHg without using antihypertensive drugs or those who have been diagnosed with hypertension or are taking antihypertensive drugs

#### 2.2.2. Measures

Fasting peripheral venous blood of the subjects was extracted, and serum after centrifugation was used for biochemical index detection. The FPG, TC, TG, LDL-C, HDL-C, and other biochemical indexes were measured by Hitachi 7600 automatic biochemical analyzer in the laboratory of the First Clinical Affiliated Hospital of Xinjiang Medical University. The quality control was qualified. White blood cells are used for genomic DNA extraction.

#### 2.2.3. Detection of 6 ABCG1 and TCF7L2 Gene Polymorphisms

The reports about human ABCG1 and TCF7L2 gene sequences were consulted, and primer was used in Premier5.0 software designed SNP site primer to be tested, using Light Cycler480 Software Setup (Roche), and was detected by fluorescence quantitative PCR. The final experimental results showed that the gene polymorphism detection results of the ABCG1 gene cg06500161 locus were all CC alleles without gene polymorphism, so the polymorphism of this gene locus was not analyzed in the future. Although rs7901695 in the promoter region of the TCF7L2 gene is a CpG-SNP site, sequencing results showed that the genotypes were TT, TC, and CC, because only 98 out of 332 samples contained the C allele, and the methylation modification was limited to cytosine in guanine–cytosine (GC) sequence. Due to the small sample size, the correlation analysis between methylation level and T2DM at this site was not conducted.

#### 2.2.4. ABCG1 Gene cg06500161 Methylation Rate Detection

Designing primers is for the promoter region of the ABCG1 gene at the cg06500161 locus. The amplification was performed by using the Applied Biosystems (ABI) (Veriti 96 well) PCR instrument, and the gene methylation level was detected by QIAGEN (Pyro Mark Q96 ID) pyrophosphate sequencer by fluorescence release and intensity. DNA quality was determined by agarose (AGAR) gel electrophoresis.

### 2.3. Intervention Measures

We conducted a health education intervention based on the content of the dietary pagoda to explore the relationship between methylation of the ABCG1 gene cg06500161 and T2DM in Uyghur. The specific steps are as follows.

Conduct diabetes health knowledge education once a month through videos, lectures, and other means, for 30–40 min. The content mainly includes (1) causes, complications, diagnosis, and harm of diabetes; (2) the benefits of reasonable dietary intake on disease and the principle of universal dietary pagoda intake; (3) the benefits of good living habits for disease prevention, such as reasonable exercise, smoking, and drinking; (4) psychological counseling, drug control, and how to conduct self-testing; and (5) follow-up questionnaire survey.

The patients who could not attend the lecture on the spot were followed up by telephone, WeChat, and face-to-face interview with health education, and the follow-up questionnaire was filled in, for 8–15 min. The patients were investigated on their health status of diet, psychology, exercise, smoking, drinking, and drinking water based on dietary pagoda. If there is a problem, communicate promptly to solve it.

### 2.4. Statistical Methods

The database was established by EpiData 3.02 software, and the data were processed by Statistical Package for the Social Sciences (SPSS) 23.0. The measurement data conforming to normal distribution were described by means ± standard deviation, and the differences between groups were analyzed by student *t* test. Data inconsistent with normal distribution were described by median (lower quartile–upper quartile), and difference comparison between two or more groups was analyzed by the Mann–Whitney *U* test. Data of categorical variables were represented by case number (component ratio), and comparison between two or more groups was analyzed by chi-square test. A univariate conditional logistic regression model was used to analyze the association between environmental factors and T2DM risk, reporting the odds ratios (ORs) and 95% confidence intervals (CIs). If *p* < 0.05, this factor was included in subsequent analysis.

The high-order interaction between gene polymorphism and methylation level and environmental factors was investigated using generalized multifactor dimensionality reduction (GMDR) software.

## 3. Results

### 3.1. Basic Information of Subjects

A total of 652 Uygur subjects were collected for this study, including 320 case and 332 control groups. There were no significant differences in gender, age, and sleep time between the case group and the control group (*p* > 0.05). However, there were significant differences in household registration type, education level, occupation, smoking, drinking, exercise, and psychological stress between the two groups (*p* < 0.05) (see Table 1).

#### 3.1.1. Comparison of Clinical Indicators Between the Case Group and Control Group

Table 2 shows the comparison of general clinical indicators between case and control groups. The results showed that the levels of BMI, SBP, FPG, TC, TG, LDL-C, and alanine aminotransferase (ALT) in the case group were higher than those in the control group, and the difference was statistically significant (*p* < 0.05). But there was no significant difference in WC, DBP, and aspartate aminotransferase (AST) between the case group and control group (*p* > 0.05). We also compared the disease distribution between the case group and the control group. The results showed that there were statistical differences in the distribution of obesity, abdominal obesity, hypertension, family history of T2DM, and dyslipidemia between the case group and the control group (*p* < 0.05) (Supporting Information 1: Table S1).

#### 3.1.2. Logistic Regression Analysis of T2DM Risk Factors

Multiple logistic regression analysis was performed with the prevalence of T2DM as dependent variables and smoking, alcohol consumption, exercise, obesity, abdominal obesity, hypertension, hypertriglyceridemia, and family history of T2DM as independent variables. The results showed that alcohol consumption, smoking, obesity, abdominal obesity, hypertension, family history of T2DM, and dyslipidemia were all risk factors for T2DM (*p* < 0.05), and exercise was protective factor for T2DM (*p* < 0.05). The assignment of variables is shown in Supporting Information 2: Table S2, and the results are shown in Supporting Information 3: Table S3.

### 3.2. Correlation Analysis Between TCF7L2 Gene Polymorphism and T2DM in Uyghur

The genetic balance test of rs7901695 locus of the TCF7L2 gene was performed in the Uyghur case group and control group. The results of the Hardy–Weinberg genetic balance test showed that there was no statistical significance in the difference of rs7901695 site (*p* > 0.05), indicating that the site had reached genetic balance in the study population and had a good population representative. The results are shown in Supporting Information 4: Table S4.

#### 3.2.1. Genotype Distribution in the Case Group and Control Group

The distribution frequencies of TT, TC, and CC genotypes at rs7901695 of the TCF7L2 gene were 56.88%, 34.06%, and 9.06% in the case group and 66.57%, 28.31%, and 5.12% in the control group, respectively. The rs7901695 locus of the TCF7L2 gene was significantly different between the two groups (*p* < 0.05). The distribution frequencies of T and C alleles at rs7901695 were 73.91% and 26.09% in the case group and 80.72% and 19.28% in the control group, respectively. The distribution of T and C alleles at rs7901695 of the TCF7L2 gene was significantly different between the two groups (*p* < 0.05). The results are shown in Table 3.

#### 3.2.2. Logistic Regression Analysis of TCF7L2 Gene Polymorphism and T2DM

Multiple logistic regression analysis was conducted with T2DM as independent variable, and the results showed that the genotype of rs7901695 of the TCF7L2 gene in the unadjusted model was correlated with the genetic susceptibility to T2DM in both additive and dominant models (*p* < 0.05). There was no significant difference between genotype and susceptibility to T2DM in the recessive model (*p* > 0.05). After adjusting for covariates, the genotype of rs7901695 of the TCF7L2 gene was correlated with genetic susceptibility to T2DM in additive, dominant, and recessive models (*p* < 0.05) (see Table 4 for details).

### 3.3. Correlation Analysis Between Methylation Level of the ABCG1 Gene and T2DM

#### 3.3.1. Basic Situation Analysis of the Case Group and Control Group Before and After Dietary Pagoda Mode Intervention

Table 5 describes the dietary changes of case and control groups before and after intervention. Before the intervention, there were significant differences in the frequency of water intake between the case group and control group (*p* < 0.05), but no significant differences in the frequency of other food intake between the case group and control group (*p* > 0.05). After intervention, there was statistical significance in the intake frequency of coarse grains, fruits, aquatic products, eggs, dairy products, soy products, nuts, edible oils, and water between the case group and control group (*p* < 0.05), but there was no statistical significance in other aspects (*p* > 0.05).

#### 3.3.2. The Methylation Level of the ABCG1 Gene in the Case Group and Control Group Before and After Dietary Pagoda Mode Intervention

Table 6 describes the methylation rate of the ABCG1 gene in case and control groups before and after intervention. The methylation level of this site was 72.11% and 70.50% in the case group and the control group, respectively. There was no significant difference in methylation level between the case group and control group (*p* > 0.05). After intervention, the methylation level of this site was 70.15% in the case group and 69.51% in the control group. There was no significant difference in methylation level between the case group and control group (*p* > 0.05). After intervention, methylation levels in both the case group and the control group were lower than before the intervention.

Table 7 analyzes the methylation level of the ABCG1 gene and the risk of T2DM. The results showed that there was no statistically significant association between the methylation level of the ABCG1 gene locus and the risk of T2DM in the unadjusted model before intervention (*p* > 0.05). After adjusting for smoking, alcohol consumption, exercise, hypertension, obesity, dyslipidemia, and FPG, there was a statistically significant association between the methylation level of the ABCG1 gene locus and the risk of T2DM (*p* = 0.015, OR: 1.023; 95% CI: 1.004~1.041). In the unadjusted model after the intervention, there was no statistically significant association between the methylation level of the ABCG1 gene locus and the risk of T2DM (*p* > 0.05). After adjusting for smoking, drinking, exercise, hypertension, obesity, dyslipidemia, and FPG, there was no statistical significance between the methylation level of the ABCG1 gene locus and the risk of T2DM (*p* > 0.05).

#### 3.3.3. Correlation Analysis Between Methylation Level of the ABCG1 Gene and Environmental Factors

There was no correlation between age, WC, and BMI and methylation rate at study sites (*p* > 0.05) (see Supporting Information 5: Table S5). However, there was a correlation between FPG and methylation at this site (*p* < 0.05). The results are shown in Supporting Information 6: Table S6. The Kruskal–Wallis rank sum test showed that there was no statistical significance in methylation between exercise and nonexercise, smoking and nonsmoking, and drinking and nondrinking (*p* > 0.05). Logistic regression analysis did not find statistically significant associations between ABCG1 gene methylation level and exercise, smoking, and alcohol consumption (*p* > 0.05) (see Supporting Information 7: Table S7).

The correlation between the methylation level of the ABCG1 gene and obesity, hypertension, and hyper-TG syndrome is shown in Supporting Information 8: Table S8. The Kruskal–Wallis rank sum test showed that the methylation rate between obesity and nonobesity, hypertension and nonhypertension, and dyslipidemia and nondyslipidemia had statistical significance (*p* < 0.05), and logistic regression analysis found that the methylation level of the ABCG1 gene was statistically significant in relation to obesity, hypertension, and dyslipidemia (*p* < 0.05), but not in relation to hypertension (*p* > 0.05).

#### 3.3.4. Analysis of Interaction Between Gene and Environmental Factors

GMDR software was used to analyze the interaction between the rs7901695 site of the TCF7L2 gene and possible environmental risk factors associated with T2DM patients by using the generalized multifactor dimension reduction method. Environmental factors included in this study included smoking, alcohol consumption, exercise, abdominal obesity, hypertension, dyslipidemia, and obesity. The results showed that the optimal fourth-order model in the gene-life behavior model was the interaction of rs7901695, smoking, alcohol consumption, and exercise, and its accuracy of the test set (0.6943) and cross consistency (10/10) were both the largest and statistically significant (*p* = 0.0010). In the gene-disease model, the optimal order model was the interaction of rs7901695, obesity, abdominal obesity, dyslipidemia, and hypertension, with a crossover agreement of 10/10 and statistical significance (*p* = 0.0010). The specific results are shown in Table 8.

GMDR software was used to analyze the interaction between the ABCG1 gene cg06500161 and possible environmental risk factors associated with T2DM patients. Environmental factors included in this study included smoking, alcohol consumption, exercise, abdominal obesity, hypertension, dyslipidemia, and obesity. The results showed that the optimal fourth-order model in the gene-life behavior model was the interaction of cg06500161, smoking, alcohol consumption, and exercise, which had the highest accuracy (0.6566) and the highest cross-agreement (10/10) and was statistically significant (*p* = 0.0010). In the gene-disease model, the optimal fifth-order model was the interaction of rs7901695, obesity, abdominal obesity, dyslipidemia, and hypertension. The accuracy of the test set (0.6744) and cross agreement (10/10) were both the largest and are statistically significant (*p* = 0.0010). The specific results are shown in Table 9.

## 4. Discussion

### 4.1. Influence of Demographic Characteristics on T2DM

This study showed that family history of T2DM is a risk factor for T2DM, which is consistent with other research results [33, 34]. The study of Su et al. also showed that compared with no family history of diabetes, people with a family history of diabetes have an increased risk of developing diabetes, and the more relatives an individual has a history of diabetes, the higher the risk of developing diabetes [35]. A study in Iran showed that people with a family history of T2DM had an increased lifetime risk of developing diabetes compared to those without a family history of T2DM [36]. Family history of T2DM increases the risk of T2DM mainly due to genetic consolidation, susceptibility genes, and lifestyle clustering [37]. Since family history is an uncontrollable factor, it is recommended to strengthen the scientific management of blood sugar, blood fat, and blood pressure and reasonable diet to prevent T2DM and reduce the incidence of T2DM in people with family history.

### 4.2. Dietary Pagoda Health Education Intervention Changes in Diet

T2DM is a metabolic disease caused by multiple factors and characterized by hyperglycemia. Current research shows that good lifestyle and behavior habits can effectively control blood glucose and prevent complications, which requires the participation of health education intervention. Studies have shown that diabetes health education is the most important way to understand diabetes-related knowledge. Through health education intervention, patients can effectively master the causes of diabetes, treatment programs, diet control, exercise programs, and various points for attention in daily life, which can effectively improve the unhealthy life behaviors of patients with diabetes and improve their health knowledge [38]. In the study of the FPG, 2 h postprandial blood glucose, and HbA1c of the experimental group were lower than before the intervention (*p* < 0.05) after 1 month of dietary nursing combined with health education intervention, and the quality of life of the patients improved after the intervention, mainly because the dietary nursing and health education intervention can improve the patients' awareness of the disease to strengthen their self-control in life [39]. The results of this study showed that before intervention, there was a significant difference between the case group and the control group only in the frequency of water intake, and there was no statistical difference in the frequency of other food intake. However, there was statistical significance in the intake frequency of coarse grains, fruits, aquatic products, eggs, dairy products, soy products, nuts, edible oils, and water between the case group and control group after the intervention. The frequency of dietary intake in all aspects increased after the intervention. These results indicate that health education intervention can help T2DM patients form more reasonable eating habits.

### 4.3. Association Between TCF7L2 Gene Polymorphism and T2DM

In 2006, first discovered and proposed the TCF7L2 gene, and the study on the association between its polymorphism and T2DM has also become a focus of attention in diabetes mellitus. Subsequently, a large number of experiments were conducted in many countries and populations, which confirmed that the TCF7 L2 gene was the strongest susceptibility gene for T2DM [40, 41]. Currently, the TCF7L2 gene variant has been replicated in several countries and is associated with T2DM. In a Moroccan study, rs7903146 (C/T) and rs12255372 (G/T) polymorphisms in the TCF7L2 gene were associated with the risk of T2DM in a Moroccan population. Studies in the Greek Cypriot population showed that the rs7901695 locus of the TCF7L2 gene was significantly associated with T2DM (OR = 1.31, 95% CI: 1.08~1.60) [42]. The meta-analysis also showed that polymorphisms at rs4506565, rs7901695, rs11196205, and rs12255372 of the TCF7L2 gene were significantly associated with T2DM susceptibility in Asians and Caucasians [43]. The above studies indicated that the rs7901695 locus of the TCF7L2 gene was significantly different in different regions of the world and correlated with T2DM. But studies in the Chinese population have had different results. A meta-analysis showed that polymorphisms of rs7901695, rs12255372, and rs7903146 in the TCF7L2 gene were associated with T2DM incidence in the Chinese population [44]. In the study of Duo et al., the rs7901695 locus of the TCF7L2 gene was associated with T2DM Uygur patients, and the C risk allele frequency was 21.9% [44]. However, in the study of Chen et al., 10 loci of the TCF7L2 gene (including rs7901695 loci) were not found to be correlated with T2DM in the Fujian Han population [45]. There was no significant correlation between rs7901695 polymorphisms of the TCF7L2 gene and T2DM susceptibility in the Han population in Inner Mongolia [44]. The results of this study showed that the TCF7L2 genotype difference was statistically significant between the case group and the control group. In the unadjusted model, the rs7901695 site was also found to be significantly correlated with T2DM disease under three different genetic models. After adjusting covariates, the rs7901695 site of the TCF7L2 gene was still correlated with genetic susceptibility to T2DM. Therefore, this study concluded that rs7901695 was correlated with T2DM incidence in Moyu County Uygur.

### 4.4. Relationship Between Methylation Level of the ABCG1 Gene and T2DM

Current studies have shown that the methylation of NLRP3, AIM2, ASC, ABCG1, TXNIP, SREBF1, and other genes is significantly correlated with T2DM, among which the methylation of ABCG1 is the strongest associated with T2DM [46, 47]. The relationship between the methylation level of the ABCG1 gene and T2DM has been paid more and more attention by scholars in recent years. The association between the methylation level of the ABCG1 gene and the risk of T2DM has been reported successively in European, Indian, and American populations, but there are few studies in the Chinese population and few reports in Moyu County, Xinjiang. A 2015 study reported the association between methylation of the ABCG1 gene and the onset of T2DM, showing that a 1% increase in the methylation level of the ABCG1 gene cg06500161 increased the risk of T2DM by 8% in the European population and 4% in Indian population [48]. A subsequent study in 2016 showed that the methylation level at cg06500161 of the ABCG1 gene was associated with an increased risk of T2DM (OR = 1.09, 95% CI: 1.02–1.16), and that the methylation level at this site was positively correlated with BMI, HbAlc, fasting insulin, and TG levels [49]. In this study, the association between the methylation level of the ABCG1 gene and T2DM was analyzed in the Uyghur population in Moyu County. After adjusting the covariable, it was found that the methylation level of cg06500161 could increase the risk of T2DM, which was consistent with the above findings.

In addition, DNA methylation levels can be affected by environmental factors and are in a dynamic state. In a longitudinal study, methylation levels of INS genes were significantly reduced after inulin intervention [50]. In another study, dietary and exercise interventions were shown to affect patterns of DNA methylation in gene regions associated with cell cycle regulation and carcinogenesis [50]. In a randomized controlled trial, higher levels of self-exercise during follow-up were associated with lower levels of GALNT9 methylation at 6 months of follow-up, suggesting that maintaining exercise behavior may have a long-term effect on methylation [51]. However, at present, there is little correlation between the changes of the ABCG1 gene methylation cg06500161 site after intervention and T2DM. Therefore, this study further analyzed the changes of ABCG1 gene methylation after intervention, and the results showed that the methylation level of ABCG1 decreased in both the case group and the control group before and after intervention. However, there was no significant difference in methylation level between the two groups, and the logistic regression after intervention showed that the methylation of the ABCG1 gene was not associated with T2DM. In a study of ABCG1 gene methylation and its dynamic changes in relation to T2DM, there was also no significant association between the dynamic changes of the ABCG1 gene cg06500161 and T2DM risk after 6 years of follow-up (*p* > 0.05). However, dynamic methylation changes of CpG at the ABCG1 site increased by ≥ 5%, and the risk of T2DM increased by 81% [52]. This suggests that we should not ignore long-term methylation changes in adults.

In addition, many risk factors affecting T2DM are related to DNA methylation, such as gender, age, smoking, and alcohol consumption. Therefore, the correlation between ABCG1 gene methylation rate and environmental factors was further analyzed, and the results showed that there was no correlation between age, WC, and BMI and ABCG1 gene methylation rate. However, some studies have shown that the ABCG1 gene methylation rate is associated with age, WC, and BMI. For example, Krause et al.'s study showed that DNA methylation of the ABCG1 gene was significantly associated with BMI [53]. In a meta-analysis, 76 differentiated CpG sites associated with T2DM were identified, and the association between DNA methylation and T2DM events was partially explained by adjustment for confounding factors such as BMI and smoking, suggesting that factors such as BMI and smoking play an important role in the association between DNA methylation and T2DM [54]. In addition, the results of this study showed that FPG, obesity, hypertension, and dyslipidemia were associated with the ABCG1 gene methylation rate and were statistically significant. This was consistent with the results of a meta-analysis, which showed that ABCG1 gene methylation was positively correlated with blood pressure, LDL level, and HbA1c level, but not with HDL level and glucose level [55]. Another study showed that methylation at cg06500 161 of the ABCG1 gene was inversely associated with HDL and TG levels [56]. Li's study also showed that obesity is associated with DNA methylation [57]. In a multiracial EWAS, ABCG1 gene methylation was associated with BMI and TG levels [58]. These studies provide a new idea for the changes of the methylation level of the ABCG1 gene.

### 4.5. Analysis of Gene–Environment Interaction

In recent years, studies on the interaction between genes and the environment have been widely applied to many diseases characterized by interactive genetic variation, such as metabolic diseases [59], nervous system diseases [60], and cardiovascular diseases [61]. Interaction refers to when two or more factors interact with each other to produce an effect that is not equal to the combined effect of their separate action, and there is interaction between the factors. When the combined effect is greater than the single effect, it is called positive interaction, indicating that two or more factors increase the effect at the same time. When the combined effect is less than the single effect, it is called negative interaction, indicating that the effect of two or more factors is weakened simultaneously. T2DM is a disease caused by both gene and environmental factors, and the interaction between gene and environmental factors plays an important role in the occurrence and development of T2DM. As T2DM is a polygenic disease, it does not follow the Mendelian pattern of inheritance and is likely to be influenced by multiple gene loci and environmental factors, resulting in high-order interactions. In 2007, GMDR was first proposed as a method to study the relationship between gene-to-gene interactions and nicotine dependence [62]. The basic principles of GMDR include cross-validation and score statistics, and the interaction between susceptibility genes and environmental factors is found by using dimension reduction strategies. In this study, GMDR software was used to analyze the interaction between TCF7L2 and ABCG1 genes and environmental factors. The results showed that rs7901695, obesity, dyslipidemia, and abdominal obesity had an interaction (*p* < 0.05). There were also interactions among rs7901695, smoking, drinking, and exercise (*p* < 0.05). At the same time, the results showed that there were interactions between the ABCG1 gene cg06500161 locus, obesity, dyslipidemia, abdominal obesity, and hypertension (*p* < 0.05), and there were also interactions between cg06500161 locus and smoking, drinking, and exercise (*p* < 0.05).

The diagnosis of T2DM has been improved with the continuous improvement of genetic technology. The occurrence and development of T2DM can be prevented through screening of T2DM susceptibility genes and changes of environmental factors (such as changing one's lifestyle). TCF7L2 and ABCG1 are currently known genes strongly associated with T2DM. With the continuous improvement of whole-genome technology, the mechanism of action of TCF7L2 and ABCG1 genes will be further understood, which is conducive to the prevention and treatment of T2DM. Xinjiang is a multiethnic region with unique demographic resources, which provide convenient sample resources for the study of genetic and environmental factors of T2DM. In the future, the polymorphism and methylation of T2DM genes among different ethnic groups can be studied, and more different gene loci can be found, providing theoretical basis for future studies on genes and T2DM.

## 5. Conclusion

In this study, 320 patients with T2DM in the case group and 332 healthy Uyghur people in the control group were analyzed through the above experiments and statistical analysis, and the following conclusions were drawn: Smoking, alcohol consumption, hypertension, family history of T2DM, dyslipidemia, and abdominal obesity are risk factors for T2DM, while physical exercise and high educational level are protective factors for T2DM. Before intervention, there was a significant difference in the frequency of water intake between the case group and the control group, and there was no statistical difference in the frequency of other food intake. After the intervention, the frequency of intake of appropriate coarse grains, fruits, aquatic products, eggs, dairy products, soy products, nuts, edible oils, and water was statistically significant between the case group and the control group, while the intake of other foods was not statistically significant. The frequency of rational dietary intake increased after the intervention. The rs7901695 locus of the TCF7L2 gene is associated with the development of T2DM in the Moyu County population, in which the C allele is a risk factor for T2DM. There was no significant difference in methylation of the ABCG1 gene between the case group and the control group before and after intervention. After adjusting for confounders before intervention, logistic regression analysis showed that ABCG1 gene methylation was a risk factor for T2DM. After adjusting for confounders, logistic regression analysis showed that methylation of the ABCG1 gene was not a risk factor for T2DM. ABCG1 gene methylation had no correlation with WC and BMI, but ABCG1 gene methylation was associated with hypertension, obesity, dyslipidemia, and fasting glucose. GMDR analysis revealed that the rs7901695 site of the ABCG1 gene and the cg06500161 site of the TCF7L2 gene interacted with hypertension, dyslipidemia, abdominal obesity, and obesity, as well as with drinking, smoking, and exercise.

## Figures and Tables

**Table 1:** Comparison of general characteristics between the case group and the control group.

**Variable**	**Case group (** **n** = 320**)**	**Control group (** **n** = 332**)**	**χ** ^2^	**p**
Gender			0.01	0.934
Male	158(49.38)	165 (49.70)		
Female	162 (50.62)	167 (50.30)		
Age			0.58	0.901
40~	81 (25.31)	88 (26.51)		
50~	136 (42.50)	132 (39.76)		
60~	78 (24.38)	83 (25.00)		
70~81	25 (7.81)	29 (8.73)		
Type of household registration			28.280	< 0.001
Rural area	169 (52.81)	107 (32.23)		
Town	151 (47.19)	225 (67.77)		
Degree of education			21.712	< 0.001
Below high school	236 (73.75)	187 (56.33)		
Senior high school or above	84 (26.25)	145 (43.67)		
Occupation			27.526	< 0.001
Farmers and herdsmen	100 (31.25)	50 (15.06)		
Private owner	77 (24.06)	95 (28.61)		
Professional technical personnel	45 (14.06)	54 (16.27)		
Cadre	32 (10.00)	52 (15.66)		
Production and transportation personnel	24 (7.50)	32 (9.64)		
Commercial personnel	17 (5.31)	27 (8.13)		
Office worker	25 (7.81)	22 (6.63)		
Drink alcohol			34.773	< 0.001
No	192 (60.00)	269 (81.02)		
Yes	128 (40.00)	63 (18.98)		
Smoking			11.656	0.001
No	253 (79.06)	295 (88.86)		
Yes	67 (20.94)	37 (11.14)		
Exercise			25.705	< 0.001
No	137 (42.81)	80 (24.10)		
Yes	183 (57.19)	252 (75.90)		
Sleep time (hours)			2.683	0.261
4–6	69 (21.56)	74 (22.29)		
7–8	207 (64.69)	226 (68.07)		
≥ 9	44 (13.75)	32 (9.64)		
Psychological pressure			6.716	0.010
No	259 (80.94)	293 (88.25)		
Yes	61 (19.06)	39 (11.75)		

**Table 2:** Comparison of clinical indicators between the case group and the control group.

**Index**	**Case group**	**Control group**	**Z**	**p**
WC (cm)	90 (79~98)	89 (78~103)	−0.061	0.951
BMI (kg/m^2^)	28.16 (24.81~29.32)	26.10 (22.76~29.34)	−3.721	< 0.001
SBP (mmHg)	130 (120~140)	120 (110~130)	−3.526	< 0.001
DBP (mmHg)	83 (70~90)	80.00 (70~90)	−1.454	0.146
FPG (mmol/L)	7.40 (7.20~7.92)	5.67 (5.19~6.30)	−20.011	< 0.001
TC (mmol/L)	4.85 (4.10~5.79)	3.93 (3.32~4.45)	−9.557	< 0.001
TG (mmol/L)	1.97 (1.56~2.57)	1.49 (1.10~2.00)	−7.965	< 0.001
HDL-C (mmol/L)	1.28 (0.93~1.87)	1.51 (1.18~2.01)	−4.407	< 0.001
LDL-C (mmol/L)	2.74 (2.21~3.26)	2.36 (2.04~2.71)	−6.741	< 0.001
AST (U/L)	26.89 (20.33~36.20)	26.35 (19.02~34.20)	−1.058	0.290
ALT (U/L)	30.47 (22.39~38.22)	27.06 (19.42~35.45)	−3.420	0.001

Abbreviations: ALT, alanine aminotransferase; AST, aspartate aminotransferase; BMI, body mass index; DBP, diastolic blood pressure; FPG, fasting blood glucose; HDL-C, high-density lipoprotein cholesterol; LDL-C, low-density lipoprotein cholesterol; SBP, systolic blood pressure; TC, total cholesterol; TG, triglyceride; WC, waist circumference.

**Table 3:** Distribution analysis of the TCF7L2 genotype and allele in the T2DM group and control group.

	**Case group**	**Control group**	**χ** ^2^	**p**
Genotype			7.795	0.020
TT	182 (56.88)	221 (66.57)		
TC	109 (34.06)	94 (28.31)		
CC	29 (9.06)	17 (5.12)		
Allele			8.651	0.003
T	473 (73.91)	536 (80.72)		
C	167 (26.09)	128 (19.28)		

**Table 4:** Association analysis between TCF7L2 gene polymorphism and the risk of T2DM.

	**Unadjusted model**			**Adjustment model**		
	**β**	**OR (95% CI)**	**p**	**β**	**OR (95% CI)**	**p**
Additive model						
TT		1.000			1.000	
TC	0.342	1.408 (1.004~1.975)	0.048	0.674	1.961 (1.009~3.812)	0.047
CC	0.728	2.071 (1.103~3.889)	0.023	1.664	5.279 (1.486~18.754)	0.010
Dominant model						
TT		1.000			1.000	
TC + CC	0.412	1.510 (1.099~2.074)	0.011	0.800	1.226 (1.167~4.247)	0.015
Recessive model						
TC + TT		1.000			1.000	
CC	0.613	1.847 (0.994~3.431)	0.052	1.301	3.671 (1.071~12.585)	0.039

*Note:* Adjustment: smoking, drinking, exercise, FPG, obesity, and high blood pressure.

**Table 5:** Comparison of diet between the case group and the control group before and after intervention.

	**Preintervention**				**Postintervention**			
	**Case**	**Control**	**χ** ^2^	**p**	**Case**	**Control**	**χ** ^2^	**p**
Moderate coarse grain			1.689	0.194			7.298	0.007
Occasionally	156 (48.75)	145 (43.67)			148 (46.25)	119 (35.84)		
Often	164 (51.25)	187 (56.33)			172 (53.75)	213 (64.16)		
Moderate potato			2.510	0.113			2.066	0.151
Occasionally	181 (56.56)	208 (62.65)			173 (54.06)	198 (59.64)		
Often	139 (43.44)	124 (37.35)			147 (45.94)	134 (40.36)		
Moderate vegetable			1.872	0.171			2.366	0.124
Occasionally	40 (12.50)	54 (16.27)			31 (9.69)	45 (13.55)		
Often	280 (87.50)	278 (83.73)			289 (90.31)	287 (86.45)		
Moderate fruit			0.108	0.742			3.099	0.009
Occasionally	107 (33.44)	107 (32.23)			94 (29.38)	119 (35.84)		
Often	213 (66.56)	225 (67.77)			226 (70.62)	213 (64.16)		
Moderate meat			0.463	0.496			0.968	0.325
Occasionally	69 (21.56)	79 (23.80)			140 (43.75)	158 (47.59)		
Often	251 (78.44)	253 (76.20)			180 (56.25)	174 (52.41)		
Appropriate number of aquatic products			0.701	0.403			6.089	0.014
Occasionally	261 (81.56)	279 (84.04)			203 (63.44)	179 (53.92)		
Often	59 (18.44)	53 (15.96)			117 (36.56)	153 (46.08)		
Moderate egg			0.032	0.857			4.402	0.036
Occasionally	178 (55.63)	187 (56.33)			167 (52.19)	146 (43.98)		
Often	142 (44.37)	145 (43.67)			153 (47.81)	186 (56.02)		
Moderate dairy			0.006	0.936			6.961	0.008
Occasionally	213 (66.56)	220 (66.27)			206 (64.38)	180 (54.22)		
Often	107 (33.44)	112 (33.73)			114 (35.62)	152 (45.78)		
Moderate soy products			1.189	0.275			4.926	0.026
Occasionally	270 (84.38)	290 (87.35)			262 (81.88)	248 (74.70)		
Often	50 (15.62)	42 (12.65)			58 (18.12)	84 (25.30)		
Moderate nuts			2.502	0.114			5.562	0.018
Occasionally	208 (65.00)	235 (70.78)			210 (65.63)	246 (74.10)		
Often	112 (35.00)	97 (29.22)			110 (34.37)	86 (25.90)		
Moderate cooking oil			0.297	0.586			7.054	0.008
Occasionally	132 (41.25)	130 (39.16)			91 (28.44)	127 (38.25)		
Often	188 (58.75)	202 (60.84)			229 (71.56)	205 (61.75)		
Moderate amount of salt			0.507	0.476			0.664	0.415
Occasionally	110 (34.38)	123 (37.05)			105 (32.81)	119 (35.84)		
Often	210 (65.62)	209 (62.95)			215 (67.19)	213 (64.16)		
Moderate water intake			6.090	0.014			8.783	0.003
Occasionally	71 (22.19)	102 (30.72)			50 (15.63)	27 (8.13)		
Often	249 (77.81)	230 (69.28)			270 (84.37)	305 (91.87)		

**Table 6:** Comparison of ABCG1 gene methylation rate between the case group and control group.

**Grouping**	**Preintervention**		**Postintervention**	
	**Case group**	**Control group**	**Case group**	**Control group**
	72.11 (64.40~82.48)	70.50 (62.53~79.43)	70.15 (65.68~80.23)	69.51 (62.03~79.94)
	−1.412		−1.334	
	0.158		0.183	

**Table 7:** Analysis of ABCG1 gene methylation levels and risk of T2DM.

**Model**	**β**	**p** ** value**	**OR (95% CI)**
Preintervention			
Model 1	0.007	0.140	1.007 (0.998~1.017)
Model 2	0.022	0.015	1.023 (1.004~1.041)
Postintervention			
Model 1	0.009	0.055	1.009 (1.000~1.017)
Model 2	−0.007	0.279	0.993 (0.980~1.006)

*Note:* Model 1: not adjusted; Model 2: smoking, alcohol consumption, exercise, hypertension, obesity, dyslipidemia, and FPG were adjusted.

**Table 8:** GMDR analysis results of the rs7901695 locus of the TCF7L2 gene interacting with environment.

**Model**	**Training set accuracy**	**Test set accuracy**	**χ** ^2^	**p**	**Cross-verify the consistency coefficient**
Gene—Disease					
Dyslipidemia	0.6518	0.6517	10	0.0010	10/10
Dyslipidemia and obesity	0.6603	0.6328	10	0.0010	10/10
Dyslipidemia, obesity, and abdominal obesity	0.6985	0.6895	10	0.0010	10/10
rs7901695, abdominal obesity, obesity, and dyslipidemia	0.7029	0.6825	10	0.0010	10/10
rs7901695, abdominal obesity, obesity, dyslipidemia, and hypertension	0.7145	0.6609	10	0.0010	10/10
Genes—Behavior of life					
Drink alcohol	0.6052	0.5928	9	0.0107	9/10
Drinking and exercise	0.6664	0.6665	10	0.0010	10/10
rs7901695, drinking, and sports	0.6928	0.6912	10	0.0010	10/10
rs7901695, drinking, smoking, and sports	0.6994	0.6943	10	0.0010	10/10

**Table 9:** GMDR analysis results of interaction between the cg06500161 locus of the ABCG1 gene and environment.

**Model**	**Training set accuracy**	**Test set accuracy**	**χ** ^2^	**p**	**Cross-verify the consistency coefficient**
Gene—Disease					
Dyslipidemia	0.6518	0.6517	10	0.0010	10/10
Obesity and dyslipidemia	0.6604	0.6257	10	0.0010	9/10
Obesity, dyslipidemia, and abdominal obesity	0.6985	0.6895	10	0.0010	10/10
cg06500161, obesity, dyslipidemia, and abdominal obesity	0.7033	0.6657	10	0.0010	9/10
cg06500161, hypertension, obesity, dyslipidemia, and abdominal obesity	0.7199	0.6744	10	0.0010	10/10
Genes—Behavior of life					
Drink alcohol	0.6052	0.5928	9	0.0107	9/10
Exercise and drinking	0.6664	0.6665	10	0.0010	10/10
cg06500161, sports, and drinking	0.6667	0.6570	10	0.0010	8/10
cg06500161, exercise, smoking, and drinking	0.6789	0.6566	10	0.0010	10/10

## Data Availability

The data are available on request from the authors. The data that support the findings of this study are available from the corresponding authors upon reasonable request.

## Supporting Information

Supporting Information 1 Table S1: Disease distribution in the case group and control group.

Supporting Information 2 Table S2: Assignment of variables.

Supporting Information 3 Table S3: Logistic regression analysis of risk factors for T2DM.

Supporting Information 4 Table S4: Hardy–Weinberg genetic balance test.

Supporting Information 5 Table S5: Correlation analysis of age, waist circumference, BMI, and methylation rate.

Supporting Information 6 Table S6: Correlation between blood lipid, fasting blood glucose, and methylation rate.

Supporting Information 7 Table S7: Association of smoking, alcohol consumption, and exercise with methylation rates.

Supporting Information 8 Table S8: Association between ABCG1 gene methylation levels and disease.
