# Supplementary material for: Association Between ABCG1/TCF7L2 and Type 2 Diabetes Mellitus: An Intervention Trial Based on a Case–Control Study
Source: J Diabetes Res. 2025 Feb 26;2025:9356676. doi: 10.1155/jdr/9356676 (PMC11986924; doi:10.1155/jdr/9356676)
Supplement: Supporting Information 1 — Table S1: Disease distribution in the case group and control group. [file 9356676.f1.docx]

# **Table S1** Disease distribution in case group and control group

| Index | Case Group | Control Group | $x^{2}$ | *P* |
| --- | --- | --- | --- | --- |
| Obesity |  |  | 41.071 | ＜0.001 |
| No | 135(42.19) | 223(67.17) |  |  |
| Yes | 185(57.81) | 109(32.83) |  |  |
| No | 115(35.94) | 164(49.40) |  |  |
| Yes | 205(64.06) | 168(50.60) |  |  |
| Hypertension |  |  | 13.363 | ＜0.001 |
| No | 150(46.87) | 203(61.14) |  |  |
| Yes | 170(53.13) | 129(38.86) |  |  |
| Family history of T2DM |  |  | 37.120 | ＜0.001 |
| No | 132(41.25) | 216(65.06) |  |  |
| Yes | 188(58.75) | 116(34.94) |  |  |
| Hypercholesterolemia |  |  | 64.009 | ＜0.001 |
| No | 186(58.13) | 286(86.14) |  |  |
| Yes | 134(41.87) | 46(13.86) |  |  |
| Hypertriglyceridemia |  |  | 47.525 | ＜0.001 |
| No | 116(36.25) | 210(63.25) |  |  |
| Yes | 204(63.75) | 122(36.75) |  |  |
| High density lipoproteinemia |  |  | 34.204 | ＜0.001 |
| No | 255(79.69) | 315(94.88) |  |  |
| Yes | 65(20.31) | 17(5.12) |  |  |
| Low high-density lipoproteinemia |  |  | 44.355 | ＜0.001 |
| No | 224(70.00) | 301(90.66) |  |  |
| Yes | 96(30.00) | 31(9.34) |  |  |
