# Supplementary material for: Association Between ABCG1/TCF7L2 and Type 2 Diabetes Mellitus: An Intervention Trial Based on a Case–Control Study
Source: J Diabetes Res. 2025 Feb 26;2025:9356676. doi: 10.1155/jdr/9356676 (PMC11986924; doi:10.1155/jdr/9356676)
Supplement: Supporting Information 2 — Table S2: Assignment of variables. [file 9356676.f2.docx]

# **Table S2** Assignment of variables

| Variable | Assignment |
| --- | --- |
| T2DM | 1= no, 2= yes |
| Type of household registration | 1= town, 2= countryside |
| Degree of education | 1= below high school, 2= above high school |
| Occupation | 1= farmers, 2= private enterprises, 3= professional and technical personnel, 4= cadres, 5= production and transportation personnel, 6= commercial catering personnel, 7= office staff |
| Psychological pressure | 1= no, 2= yes |
| Sleep time | 1=7~8 hours, 2= < 7 hours, 3= > 8 hours |
| Exercise | 1= no, 2= yes |
| Smoking | 1= no, 2= yes |
| Drink alcohol | 1= no, 2= yes |
| Obesity | 1= no, 2= yes |
| Abdominal obesity | 1= no, 2= yes |
| Family history of T2DM | 1= no, 2= yes |
| Hypertension | 1= no, 2= yes |
| Hypertriglyceridemia | 1= no, 2= yes |
| Hypercholesterolemia | 1= no, 2= yes |
| High density lipoproteinemia | 1= no, 2= yes |
| Low high-density lipoproteinemia | 1= no, 2= yes |
