# Supplementary material for: Association Between ABCG1/TCF7L2 and Type 2 Diabetes Mellitus: An Intervention Trial Based on a Case–Control Study
Source: J Diabetes Res. 2025 Feb 26;2025:9356676. doi: 10.1155/jdr/9356676 (PMC11986924; doi:10.1155/jdr/9356676)
Supplement: Supporting Information 3 — Table S3: Logistic regression analysis of risk factors for T2DM. [file 9356676.f3.docx]

# **Table S3** Logistic regression analysis of risk factors for T2DM

| Variable | *β* | *Wald* | *P* | *OR* | 95%*CI* |
| --- | --- | --- | --- | --- | --- |
| Type of household registration |  |  |  |  |  |
| Town |  |  |  | 1.000 |  |
| Rural area | 0.426 | 3.367 | 0.067 | 1.530 | 0.971~2.411 |
| Degree of education |  |  |  |  |  |
| Below high school |  |  |  | 1.000 |  |
| Senior high school or above | -1.056 | 17.684 | ＜0.001 | 0.348 | 0.213~0.569 |
| Occupation |  |  |  |  |  |
| Farmers and herdsmen |  |  |  | 1.000 |  |
| Private owner | -0.834 | 7.548 | 0.006 | 0.434 | 0.240~0.787 |
| Professional technical personnel | -0.340 | 0.753 | 0.386 | 0.712 | 0.331~1.533 |
| cadre | -0.854 | 4.402 | 0.036 | 0.426 | 0.192~0.945 |
| Production and transportation personnel | -0.559 | 1.750 | 0.186 | 0.572 | 0.250~1.308 |
| Commercial personnel | -1.487 | 11.365 | 0.001 | 0.226 | 0.095~0.537 |
| Office worker | -0.190 | 0.179 | 0.672 | 0.827 | 0.343~1.994 |
| Psychological pressure |  |  |  |  |  |
| No |  |  |  | 1.000 |  |
| Yes | 0.201 | 0.444 | 0.505 | 1.223 | 0.676~2.212 |
| Sleep time |  |  |  |  |  |
| 7 to 8 hours |  |  |  | 1.000 |  |
| < 7 hours | -0.325 | 1.557 | 0.212 | 0.723 | 0.434~1.204 |
| > 8 hours | -0.243 | 0.463 | 0.496 | 0.784 | 0.389~1.580 |
| Exercise |  |  |  |  |  |
| No |  |  |  | 1.000 |  |
| Yes | -0.803 | 10.140 | 0.001 | 0.448 | 0.273~0.734 |
| Smoking |  |  |  |  |  |
| No |  |  |  | 1.000 |  |
| Yes | 1.112 | 13.366 | ＜0.001 | 3.041 | 1.675~5.521 |
| Drink alcohol |  |  |  |  |  |
| No |  |  |  | 1.000 |  |
| Yes | 0.999 | 14.510 | ＜0.001 | 2.716 | 1.624~4.542 |
| Family history of T2DM |  |  |  |  |  |
| No |  |  |  | 1.000 |  |
| Yes | 1.132 | 21.381 | ＜0.001 | 3.101 | 1.920~5.011 |
| Obesity |  |  |  |  |  |
| No |  |  |  | 1.000 |  |
| Yes | 0.706 | 9.665 | 0.002 | 2.026 | 1.298~3.162 |
| Abdominal obesity |  |  |  |  |  |
| No |  |  |  | 1.000 |  |
| Yes | 0.913 | 11.796 | 0.001 | 2.491 | 1.480~4.195 |
| Hypertension |  |  |  |  |  |
| No |  |  |  | 1.000 |  |
| Yes | 0.570 | 7.269 | 0.007 | 1.769 | 1.168~2.677 |
| Hypertriglyceridemia |  |  |  |  |  |
| No |  |  |  | 1.000 |  |
| Yes | 1.132 | 28.891 | ＜0.001 | 3.100 | 2.052~4.684 |
| Hypercholesterolemia |  |  |  |  |  |
| No |  |  |  | 1.000 |  |
| Yes | 1.261 | 21.023 | ＜0.001 | 3.529 | 2.059~6.051 |
| High-density lipoproteinemia |  |  |  |  |  |
| No |  |  |  | 1.000 |  |
| Yes | 0.958 | 6.734 | 0.009 | 2.607 | 1.264~5.377 |
| Low high-density lipoproteinemia |  |  |  |  |  |
| No |  |  |  | 1.000 |  |
| Yes | 1.081 | 12.466 | ＜0.001 | 2.948 | 1.618~5.373 |
