# Supplementary material for: Association Between ABCG1/TCF7L2 and Type 2 Diabetes Mellitus: An Intervention Trial Based on a Case–Control Study
Source: J Diabetes Res. 2025 Feb 26;2025:9356676. doi: 10.1155/jdr/9356676 (PMC11986924; doi:10.1155/jdr/9356676)
Supplement: Supporting Information 4 — Table S4: Hardy–Weinberg genetic balance test. [file 9356676.f4.docx]

# **Table S4** Hardy-Weinberg genetic balance test

| Genotype | Case Group | | Control Group | |
| --- | --- | --- | --- | --- |
|  | Observed Value | Expected Value | Observed Value | Expected Value |
| TT | 182 | 197.8 | 221 | 205.2 |
| TC | 109 | 99.6 | 94 | 103.4 |
| CC | 29 | 22.6 | 17 | 23.4 |
| $\chi^{2}$ | 1.752 | | 1.911 | |
| *P* | 0.416 | | 0.385 | |
