# Supplementary material for: Association Between ABCG1/TCF7L2 and Type 2 Diabetes Mellitus: An Intervention Trial Based on a Case–Control Study
Source: J Diabetes Res. 2025 Feb 26;2025:9356676. doi: 10.1155/jdr/9356676 (PMC11986924; doi:10.1155/jdr/9356676)
Supplement: Supporting Information 5 — Table S5: Correlation analysis of age, waist circumference, BMI, and methylation rate. [file 9356676.f5.docx]

# **Table S5** Correlation analysis of age, waist circumference, BMI and methylation rate

|  | Age | | Waist Circumference | | BMI | |
| --- | --- | --- | --- | --- | --- | --- |
|  | r | *P* | r | *P* | r | *P* |
| CpG (%) | 0.029 | 0.459 | -0.021 | 0.598 | 0.023 | 0.559 |
