# Supplementary material for: Association Between ABCG1/TCF7L2 and Type 2 Diabetes Mellitus: An Intervention Trial Based on a Case–Control Study
Source: J Diabetes Res. 2025 Feb 26;2025:9356676. doi: 10.1155/jdr/9356676 (PMC11986924; doi:10.1155/jdr/9356676)
Supplement: Supporting Information 6 — Table S6: Correlation between blood lipid, fasting blood glucose, and methylation rate. [file 9356676.f6.docx]

# **Table S6** Correlation between blood lipid, fasting blood glucose and methylation rate

|  | TC | | TG | | HDL | | LDL | | GLU | |
| --- | --- | --- | --- | --- | --- | --- | --- | --- | --- | --- |
|  | r | *P* | r | *P* | r | *P* | r | *P* | r | *P* |
| CpG (%) | 0.064 | 0.104 | 0.007 | 0.860 | -0.036 | 0.357 | 0.007 | 0.857 | -0.153 | 0.000 |
