# Supplementary material for: Association Between ABCG1/TCF7L2 and Type 2 Diabetes Mellitus: An Intervention Trial Based on a Case–Control Study
Source: J Diabetes Res. 2025 Feb 26;2025:9356676. doi: 10.1155/jdr/9356676 (PMC11986924; doi:10.1155/jdr/9356676)
Supplement: Supporting Information 7 — Table S7: Association of smoking, alcohol consumption, and exercise with methylation rates. [file 9356676.f7.docx]

# **Table S7** Association of smoking, alcohol consumption and exercise with methylation rates

| Variable | Methylation rate（%） | **P* | ^#^β | ^#^*P* | ^#^*OR*（95%*CI*） |
| --- | --- | --- | --- | --- | --- |
| Exercise |  | 0.313 | 0.003 | 0.539 | 1.003（0.993~1.014） |
| Yes | 71.61（63.86~81.40） |  |  |  |  |
| No | 70.29（63.80~78.91） |  |  |  |  |
| Smoking |  | 0.472 | -0.003 | 0.662 | 0.997（0.984~1.010） |
| Yes | 69.86（62.58~80.19） |  |  |  |  |
| No | 71.48（63.93~81.02） |  |  |  |  |
| Drink alcohol |  | 0.413 | -0.004 | 0.499 | 0.996（0.985~1.007） |
| Yes | 70.04（62.49~80.31） |  |  |  |  |
| No | 71.56（63.97~81.12） |  |  |  |  |

Methylation levels are expressed as median (lower quartile - upper quartile)

* Kruskal-Walli’s rank sum test was used

logistic regression model was used to adjust for obesity, abdominal obesity, hypertension, family history of T2DM, FPG and dyslipidemia
