# Supplementary material for: Association Between ABCG1/TCF7L2 and Type 2 Diabetes Mellitus: An Intervention Trial Based on a Case–Control Study
Source: J Diabetes Res. 2025 Feb 26;2025:9356676. doi: 10.1155/jdr/9356676 (PMC11986924; doi:10.1155/jdr/9356676)
Supplement: Supporting Information 8 — Table S8: Association between ABCG1 gene methylation levels and disease. [file 9356676.f8.docx]

# **Table S8** Association between ABCG1 gene methylation levels and disease

| Variable | Methylation rate（%） | **P* | ^#^*β* | ^#^*P* | ^#^*OR*（95%*CI*） |
| --- | --- | --- | --- | --- | --- |
| Obesity |  | 0.004 | 0.015 | 0.004 | 1.015（1.005~1.026） |
| Yes | 72.66（66.55~82.42） |  |  |  |  |
| No | 69.48（61.19~82.42） |  |  |  |  |
| Abdominal obesity |  | 0.514 | 0.005 | 0.329 | 1.005（0.995~1.016） |
| Yes | 71.38（64.66~80.38） |  |  |  |  |
| No | 70.73（62.18~81.40） |  |  |  |  |
| Hypertension |  | 0.001 | 0.015 | 0.003 | 1.016（1.005~1.026） |
| Yes | 73.01（65.23~85.48） |  |  |  |  |
| No | 69.93（62.38~78.21） |  |  |  |  |
| Dyslipidemia |  | 0.007 | 0.014 | 0.007 | 1.014（1.004~1.025） |
| Yes | 72.46（64.44~82.45） |  |  |  |  |
| No | 69.58（61.38~76.75） |  |  |  |  |

Note: Methylation rate is expressed as median (lower quartile - upper quartile)

* Kruskal-Walli’s rank sum test was used

Logistic regression model was used to adjust for family history of T2DM, smoking, alcohol consumption, exercise, and FPG.
